# Acute Kidney Injury Associated With Dehydration Protocol Used by Combat Sports Athletes

**DOI:** 10.1155/tsm2/2946676

**Published:** 2026-02-02

**Authors:** Antônio André Jarsen Pereira, Lucas Magri, Marina de Moura Bello, Andréia Cristina Febba Gomes, Carlos Eduardo Neves Amorim, Lilian Caroline Gonçalves de Oliveira, Nestor Schor, Dulce Elena Casarini

**Affiliations:** ^1^ Nephrology Division, Medicine Department, Escola Paulista de Medicina, Universidade Federal de São Paulo, Rua Pedro de Toledo, 669, São Paulo, 04039-032, Brazil, unifesp.br; ^2^ Post-Graduation Program in Physical Education, Universidade Federal Do Maranhão, Avenida Dos Portugueses, 1966, São Luis, 65080-805, Brazil, ufma.br

**Keywords:** acute kidney injury, combat sports, dehydration, Mixed Martial Arts, Muay Thai

## Abstract

The increase in combat sports practice and the creation of weight divisions for fairer competitions led to dehydration practice as a strategy of inclusion in inferior divisions. However, this technique can damage kidney and heart functions due to alterations in blood volume. This study evaluated the acute effects of weight loss through dehydration on the kidney function of Mixed Martial Arts and Muay Thai fighters. The sample was composed of 30 athletes of both Mixed Martial Arts (*n* = 15) and Muay Thai (*n* = 15) fighters. Both groups went through two protocols for collecting data about the athlete’s profile, vital signs, and urinary and blood samples in three different moments: before weigh‐in, official weigh‐in day, and fight day. The athletes’ profiles and the dehydration methods employed were found to be consistent with those reported in the literature. The participants lost weight 1 month before the fight and had alterations that developed into glycosuria, leukocyturia, and proteinuria noted on both official weigh‐in and combat days. Proteinuria and high creatinine depuration suggest acute kidney damage with an increase in filtration rate due to dehydration. As shown, there is a necessity for proper athlete orientation regarding dehydration and possible damage to the body’s physiological integrity and sport performance, and developing a guide on more appropriate weight control protocols that do not put athletes’ health at risk should be established and publicized.

## 1. Introduction

Although commonly stated that it hailed from Japan, some authors state that the soft art, Jiu Jitsu, was brought to China from India, thanks to the monks, as it was created with a defensive nature through the applications of physics laws, based on the human body lever systems, the relation between force and body balance focused on the gravity center, and human body vital point studies [1].

An increase in the participation in martial arts can be observed recently, with a predilection for Jiu‐Jitsu, Muay Thai (MUAY), and Mixed Martial Arts (MMA). The Ultimate Fighting Championship (UFC), a well‐known worldwide event in the sports community, hosts a show that involves MMA practice in the combat scenario, contemplating a combination of different sports in the fight modality, as one [2–5].

With media contributing to UFC event divulgation and the MMA’s interested public growth, with less interest related to MUAY, which is justified by the much lesser research in the area, a critical approach was introduced to the modality for applying rules that would guarantee less disparity among competitors, becoming a court‐dealt issue, that interfered with both the technique popularity and the event. This way, to mitigate those problems, weight divisions and self‐protection equipment use were included, while more traumatic move techniques were abolished [6].

The weight division rule ensures that the official weigh‐in conference occurs before the competition, usually 24 h before the official fight, for a fairer combat. However, intending to get sorted into lower categories, many athletes go through the process of hydric loss, evolving into dehydration hours before the combats, a technique known as rapid weight loss (RWL) [7, 8].

For inducing RWL, according to Barley and colleagues [9], fighters of different modalities go through strategies, such as food and liquid intake restriction, sauna and/or hot and humid places, increased vigorous exercise practice with plastic clothing, and use of diuretics [10–12].

MMA competitors are mostly RWL supporters as a strategy for quick body weight loss before the official weigh‐in. Still, the induced dehydration may compromise the competitor’s performance, as well as lead to physiological damages [13]. Moreover, due to induced dehydration, the modality has faced fatalities such as death and Acute Kidney Injury (AKI) [14, 15].

Induced dehydration results in a significant loss of fluid from blood plasma, leading to hypovolemia. This condition can trigger a range of alterations, including cardiovascular, immunologic, cognitive, perceptive, and kidney dysfunction, among other complications [16–18]. These effects may contribute to the development of AKI and, therefore, increase the risk of progressing to Chronic Kidney Disease (CRD) [19–22].

In this context, it is understood that the majority of body weight loss in fighters aiming to qualify for lower weight categories comes from the acute loss of body fluids, mainly blood plasma. This leads to an increase in osmolarity due to hypovolemia, which may cause physiological alterations, as morphological and/or functional kidney and heart compromising potential in this population.

Assuming that about 65% of the human body is made up of water [23], and that intentional dehydration in combat athletes is related to quick body weight loss, this study’s main objective was to evaluate the dehydration influence on the kidney function of combat sports athletes during the pre‐weigh‐in, weigh‐in day, and competition days.

## 2. Materials and Methods

All procedures were agreed with the Helsinki declaration of 2024 and its amendments and were approved by the institutional ethics committee on human experimentation from Federal University of São Paulo (UNIFESP) under the register number 0272/2018. All athletes were informed of this study’s objectives and possible risks and accepted to participate as volunteers after signing the Free and Informed Consent Form (FICF).

Thirty (30) male participants were included in the sample, ages ranging from 18 to 37 years old, with a minimum sport practice time of 6 months, during competitive period, either or not follower of the dehydration process for body weight loss. The participants were divided into two groups: the MUAY group (MUAYG, *n* = 15) and the MMA group (MMAG, *n* = 15).

Data and sample collections were organized in two protocols. Protocol A (first assessment) consisted of FICF and questionnaire applications and body weight evaluation. Protocol B (second and third assessments) consisted of a follow‐up questionnaire application: blood pressure (BP) assessment, heart rate (HR), body weight, urine sample collection, urine color classification, and blood sample collection.

Both groups underwent 3 assessments in 60 days: precompetition/about 45 days before the combat (first assessment), on the official weigh‐in day (second assessment), and combat day (third assessment). Protocol A was applied only on the first assessment, while Protocol B was applied in the three assessments.

## 3. Data Collection Procedures

### 3.1. Application of the Athlete’s Profile Questionnaire (Included as Supporting Information)

During the first stage of this study, all volunteers provided information about their athletic profile, preexisting comorbidities, family pathological history, and the relation between their precompetition behavior and the possible acute body weight loss through dehydration.

### 3.2. BP Assessment

BP was assessed by the investigator using a sphygmomanometer (aneroid type, Premium, Brazil) and a stethoscope (Rappaport, Brazil). During the BP assessment, the participants were instructed as follows: to arrive at the training center and remain seated on a chair with backrest for 5–10 min before starting; remain upright with erect posture; let the left arm available for positioning the sphygmomanometer cuff approximately 3 cm above the cubital fossa in supination and then rest the arm on a surface higher than the heart line; and remain silent during the procedure, with the arm relaxed and free from movements. Three measurements were taken by the investigator with a one‐minute interval between them. The first measure was disregarded, and the second and third averaged; in case of discrepancy > 5 mm/Hg, a fourth measurement was required, and the closest measurements were averaged.

### 3.3. HR Assessment

For assessing the HR, a pulse oximeter (Geratherm, Germany) was used, with a photoelectric evaluation method, SpO2 range: 70%—90%, and pulse: 30–235 bpm.

### 3.4. Body Weight (BW) Assessment

The participants were instructed to wear only bathing suits or swimming trunks and to remain in the anatomic position for body weighing with a portable digital scale (Premium, Brazil) placed on a wooden floor.

### 3.5. Urine Collection

The participants were oriented to discard the first urine stream and then collect the rest in a universal urine collector (J. Prolab, Brazil), properly identified and provided by the researcher. The samples were then kept in a 4°C thermal box until analysis.

Approximately half of the volume was destined for semiquantitative chemical analysis, while the other half was centrifuged at 1060 rcf for 10 min at 4°C for dosage of microalbuminuria (turbidimetric system); urea (colorimetric enzymatic system); creatinine, magnesium, and calcium (colorimetric system); and potassium and sodium (enzymatic system). The ratio between urea and creatinine (U/Cr); fractional excretion of sodium (FENa), obtained through the equation ([urinary sodium/serum sodium]/[urinary creatinine/serum creatinine] *×* 100); and fractional excretion of potassium (FEK), obtained through the equation ([urinary potassium/serum potassium]/[urinary creatinine/serum creatinine] *×* 100), was calculated using Microsoft Excel 2010. The volunteers’ hydration condition was assessed through the urine color table using the ComburTest Dabe‐Behring, allowing their classification as well hydrated, slightly dehydrated, moderately dehydrated, and severely dehydrated.

The urine physicochemical analysis was performed using ComburTest Dabe‐Behring polyelectrolytes strips for semiquantitative detection of proteins, glucose, nitrite, bilirubin, pH, ketonic bodies, leukocytes, blood, and urobilinogen in noncentrifuged urine samples.

### 3.6. Blood Collection

Approximately 10 mL of venous blood was collected in K3 Vacuette tubes containing EDTA, with proper identification, and were kept inside a 4°C thermal box until centrifugation. The samples were centrifuged at 1060 rcf at 4°C for 10 min, and the supernatant was collected and kept in vials at −20°C for later analysis of creatinine and urea.

A colorimetric system was used for serum creatinine quantification, and a colorimetric enzymatic system for urea, based on the Labtest commercial kits, and the Labmax equipment were used for reading at 37°C and a wavelength of 505 nm.

The values of the U/Cr ratio and the estimated glomerular filtration rate were obtained using the CKD‐EPI equation and expressed in mg/dL.

### 3.7. Statistical Analysis

The statistics were done using the IBM SPSS Statistics v. 20 software. The results for the continuous variables of the present study were expressed as mean and standard deviation.

The Kolmogorov–Smirnov test was conducted as a normality test to analyze the distribution of the values found. Once the normal distribution for all the variables, the ANOVA test with repeated measures was used in the comparison of the continuous variables in different moments of the study and the paired T‐student test was used in the comparison of data in two different moments of the study. For the categorical variable statistical analysis, the Fisher exact test was used. For answering correlations between specific variables, the Pearson test was used. It was considered statistically significant data with 5% significance level.

## 4. Results

The mean age was similar for MMAG (26 ± 4.97 years old) and MUAYG (25 ± 4.95 years old). Most athletes had between 2 and 3 years of competitive experience, characterizing a phase of sporting maturation.

The initial BW mean was 74.7 ± 6.68 kg for MMAG and 70.6 ± 8.33 kg for MUAYG, and on the second assessment, the MMAG lost 10 ± 5.95% of BW, while the MUAYG lost 7.7 ± 2.92% of BW (Table 1), but both groups ended the study recovering weight by the third assessment (MMAG: 6 + 3.06 kg increase; MUAYG: 7.5 + 3.63 kg increase), recovering or gaining more weight than before (Figure 1).

**Table 1:** Comparison between Mixed Martial Arts group (MMAG) and Muay Thai group (MUAYG) mean body weights on the three evaluations.

	MMAG	MUAYG
Body weight approximately 45 days before the fight (kg)	74.7 ± 6.68	70.6 ± 8.33
Body weight on the official weigh‐in day (kg)	67.3 ± 8.40	64.9 ± 6.40
Body weight on the combat day (kg)	73.3 ± 7.70	69.8 ± 7.17
Body weight loss between the 1^st^ and 2^nd^ assessments (%)	10 ± 5.95	7.7 ± 2.92
Body weight loss between the 2^nd^ and 3^rd^ assessments (%)	9.3 ± 4.98	7.5 ± 3.63
Mean difference between the 1^st^ and 2^nd^ assessments (kg)	7.3 ± 4.18	5.6 ± 3.08
Mean difference between the 2^nd^ and 3^rd^ assessments (kg)	6 ± 3.06	4.9 ± 2.30

Figure 1Body weight of the MMAG and MUAYG group across the three assessments. (a) MMAG body weight means in the 1^st^, 2^nd^, and 3^rd^ assessments. (b) MUAYG body weight means in the 1^st^, 2^nd^, and 3^rd^ assessments. ^∗^ *p* < 0.001*—*HR 1^st^ assessment vs. 2^nd^ assessment (MMAG). Values are expressed as mean ± SD. Repeated measures ANOVA followed by post hoc test.

**Figure:**
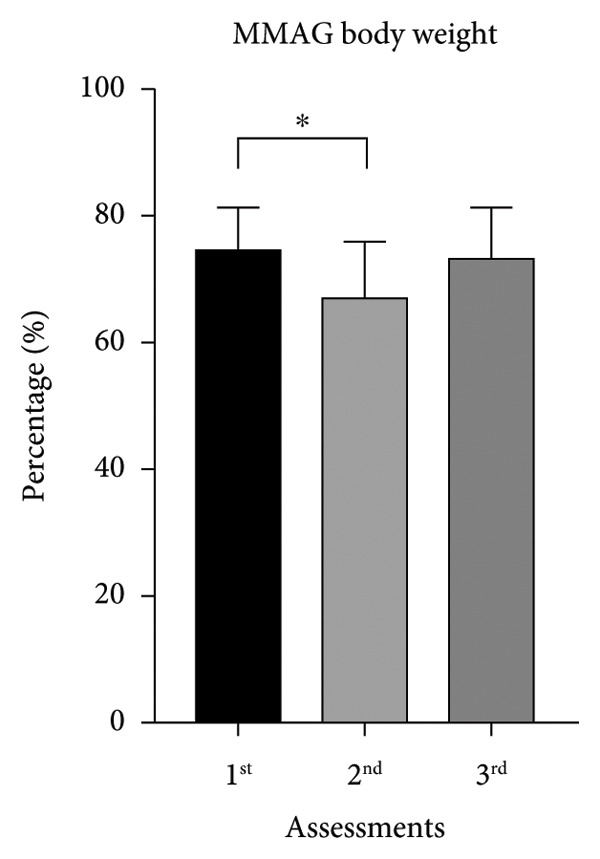
(a)

**Figure:**
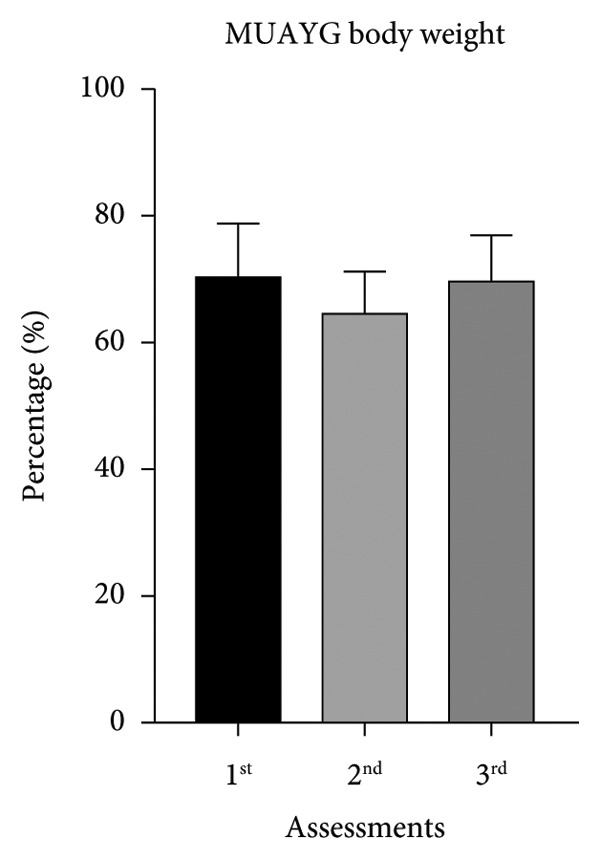
(b)

Both groups (80% of MMAG; 100% of MUAYG) began their weight loss activities between 8 and 29 days before the official weigh‐in and used the same food and liquid restrictions, sweating induced by physical activity, vigorous exercising with plastic clothing, and/or in hot and humid places. It is important to highlight that, in the present study, there was no control over the use of laxatives or diuretics, since the observed competitions did not involve antidoping monitoring. Therefore, athletes had full autonomy to decide whether or not to adopt such practices. Diuretic ingestion was reported by 100% of MMAG volunteers and by 40% of MUAYG volunteers. This pattern was reflected in the hydration status assessed in the second session, when 94% of MMAG athletes were dehydrated or severely dehydrated, compared with 100% of MUAYG athletes classified as severely dehydrated. Nevertheless, hydration was restored by the third session (87% and 100%, respectively).

It is well documented that combat sport athletes commonly implement rapid weight reduction strategies, primarily through methods that induce fluid loss and consequently acute dehydration, in order to comply with weight‐class requirements. Following a weigh‐in, athletes typically engage in rapid body mass recovery protocols, characterized by increased fluid and nutritional intake. These practices are designed to restore body weight and, theoretically, facilitate the transition from a dehydrated to a rehydrated state before competition. This study did not evaluate the specific rehydration strategies implemented by athletes between the second assessment (weigh‐in) and the third assessment (competition). It is likely that individualized approaches were employed during this interval, which may partly account for the differences in hydration status observed between the groups.

MMAG’s HR mean on the first and second assessments was similar to MUAYG’s HR, with a significant difference observed when comparing the second and third assessments (Figure 2).

Figure 2Heart rate (HR) of the MMA and MUAY group across the three assessments. (a) MMAG heart rate means in the 1^st^, 2^nd^, and 3^rd^ assessments. (b) MUAYG heart rate means in the 1^st^, 2^nd^, and 3^rd^ assessments. ^∗^ *p* < 0.001*—*HR 1^st^ assessment vs. 3^rd^ assessment (MMAG and MUAYG) and 2^nd^ assessment vs. 3^rd^ assessment (MMAG and MUAYG). Values are expressed as mean ± SD. Repeated measures ANOVA followed by post hoc test.

**Figure:**
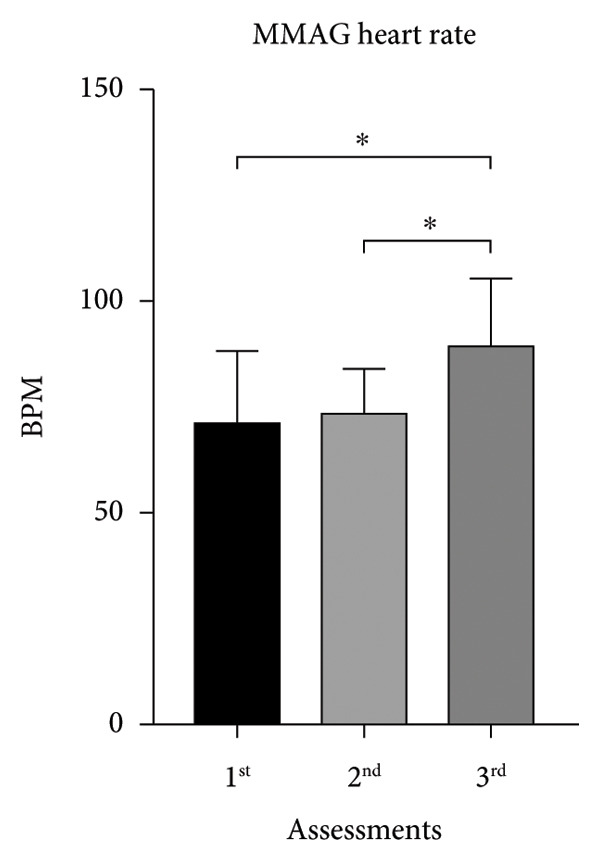
(a)

**Figure:**
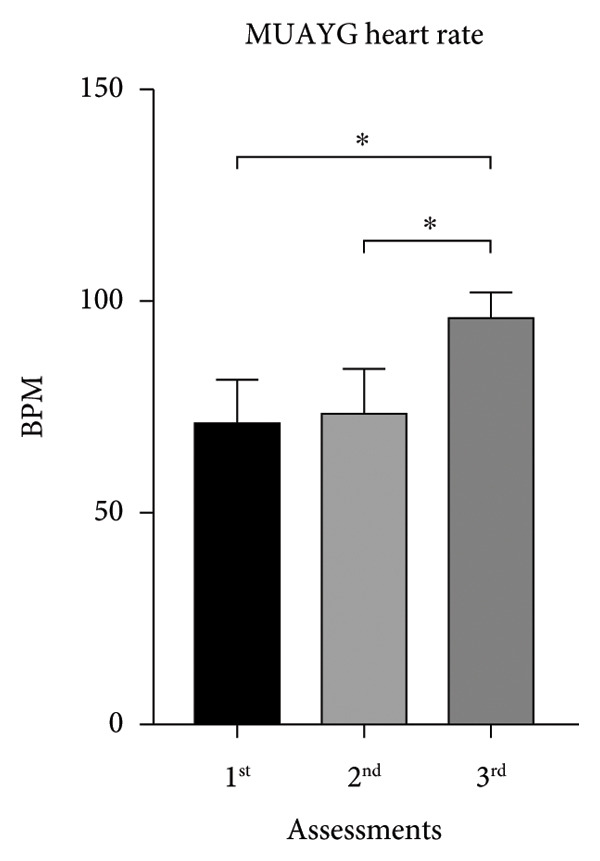
(b)

The systolic and diastolic arterial pressures (SAP and DAP) showed significant differences in both groups in the second and third assessments, except for MUAYG’s DAP (Figure 3).

Figure 3Systolic arterial pressure (SAP) and diastolic arterial pressure (DAP) of the MMA and MUAY groups across the three assessments. (a) MMAG systolic arterial pressure means in the 1^st^, 2^nd^, and 3^rd^ assessments. (b) MMAG diastolic arterial pressure means in the 1^st^, 2^nd^, and 3^rd^ assessments. (c) MUAYG systolic arterial pressure means in the 1^st^, 2^nd^, and 3^rd^ assessments. (d) MUAYG diastolic arterial pressure means in the 1^st^, 2^nd^, and 3^rd^ assessments. SAP: systolic arterial pressure; DAP: diastolic arterial pressure. ^∗^ *p* < 0.001*—*SAP 1^st^ assessment vs. 3^rd^ assessment (MMAG and MUAYG), 2^nd^ 1assessment vs. 3^rd^ assessment (MMAG and MUAYG), and DAP 2^nd^ assessment vs. 3^rd^ assessment (MMAG). Values are expressed as mean ± SD. Repeated measures ANOVA followed by post hoc test.

**Figure:**
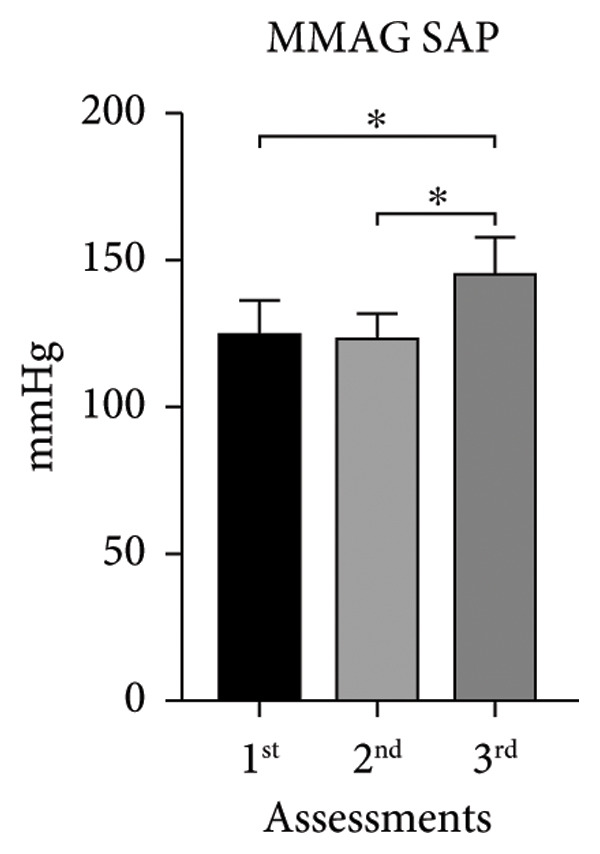
(a)

**Figure:**
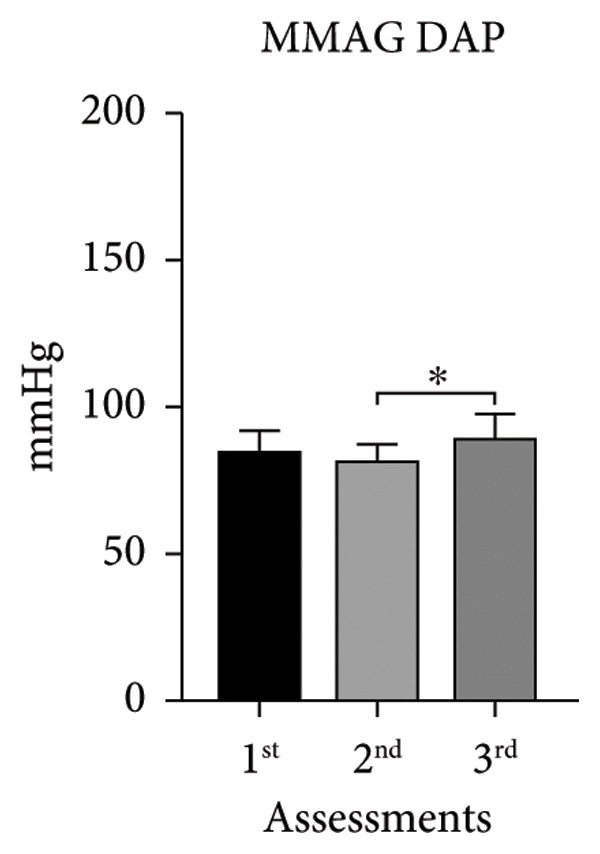
(b)

**Figure:**
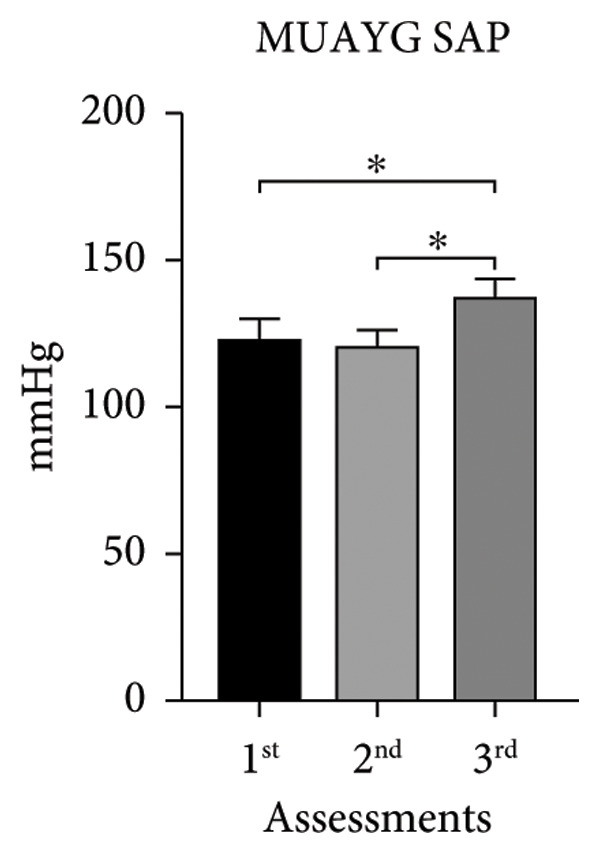
(c)

**Figure:**
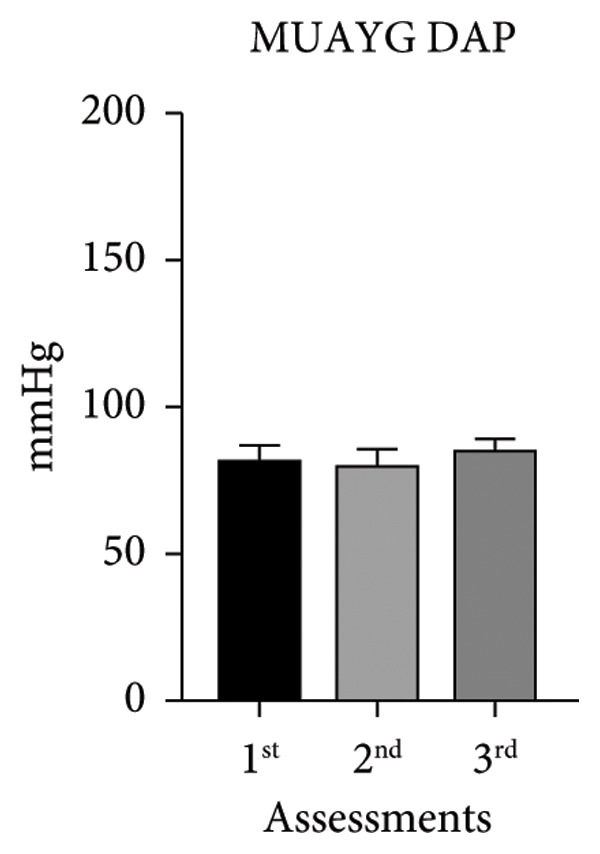
(d)

### 4.1. Urine and Blood Analysis

Both MMAG and MUAYG groups presented hematuria and nitrites on the second and third assessments, which were negative on the first assessments. Although both groups showed high proteinuria levels on the first and second assessments, it appeared in only 40% and 13% of MMAG and MUAYG, respectively, by the third assessments. Leukocyturia was not detected on the combat day, despite being shown on the previous dates.

Throughout the study, MMAG blood creatinine increased about 21% between the first and second assessments, associated with body weight reduction, but the increment was reversed by the third assessments. The same happened in the MUAYG, although the difference was not statistically significant (Figure 4).

Figure 4Serum creatinine of the MMA and MUAY groups across the three assessments. (a) MMAG serum creatinine means in the 1^st^, 2^nd^, and 3^rd^ assessments. (b) MUAYG serum creatinine means in the 1^st^, 2^nd^, and 3^rd^ assessments. ^∗^ *p* = 0.001*—* 1^st^ assessment vs. 2^nd^ assessment and ^∗^ *p* = 0.002 2^nd^ assessment vs. 3^rd^ assessment (MMAG). Values are expressed as mean ± SD. Repeated measures ANOVA followed by post hoc test.

**Figure:**
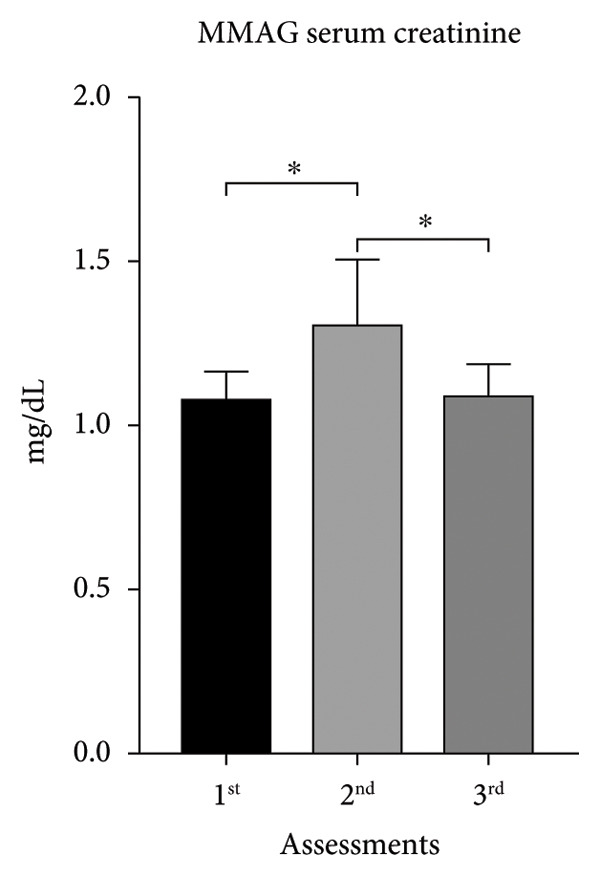
(a)

**Figure:**
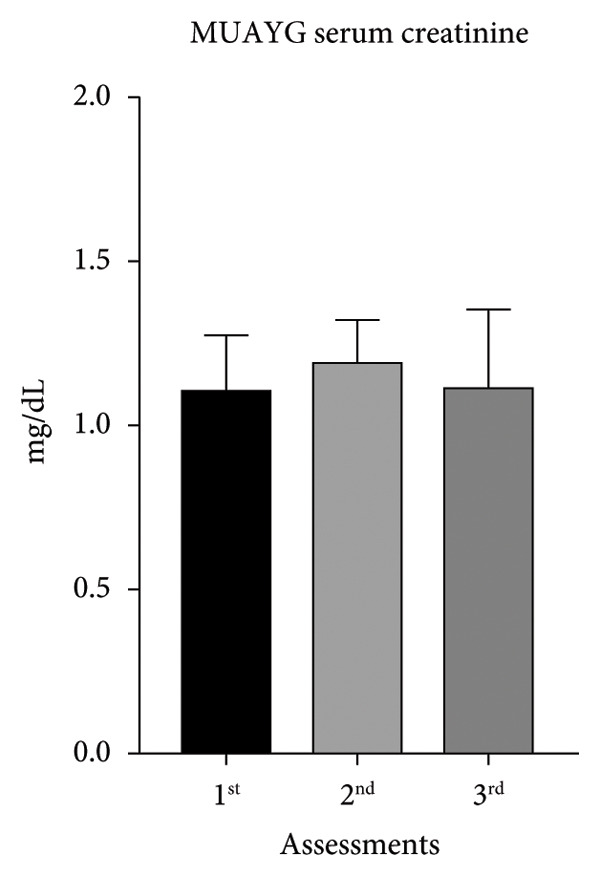
(b)

Following the same profile as creatinine, urea increased in both groups between the first two evaluations (MMAG: 26%; MUAYG: 7%), showing a decrease between the last ones (MMAG: 17%; MUAYG: 14%) (Figure 5). Creatinine and urea level fluctuations may be related to BW loss or gain, as well as fluid loss, which impacts kidney function, leading to hematuria, nitrites, and leukocyturia in the urine. After fluid recovery, there was a significant reduction in proteinuria, especially in the GMUAY group, in which 87% of athletes achieved normalization of this marker, compared with 13% in the GMMA group (Table 2). These findings reinforce the idea that rapid weight gain in fighters can induce transient changes in kidney function, and prolonged monitoring is essential to differentiate reversible adaptations from potential progression to CKD (Table 2).

Figure 5Serum urea of the MMA and MUAY groups across the three assessments (a) MMAG serum urea means in the 1^st^, 2^nd^, and 3^rd^ assessments. (b) MUAYG serum urea means in the 1^st^, 2^nd^, and 3^rd^ assessments. ^∗^ *p* < 0.05. Values expressed as mean ± ANOVA repeated measures SD + post hoc. ^∗^ *p* < 0.05. Values expressed as mean ± ANOVA repeated measures SD + post hoc. ^∗^ *p* = 0.001*—*1^st^ assessment vs. 2^nd^ assessment and ^∗^ *p* = 0.002 2^nd^ assessment vs. 3^rd^ assessment (MMAG). Values are expressed as mean ± SD. Repeated measures ANOVA followed by post hoc test.

**Figure:**
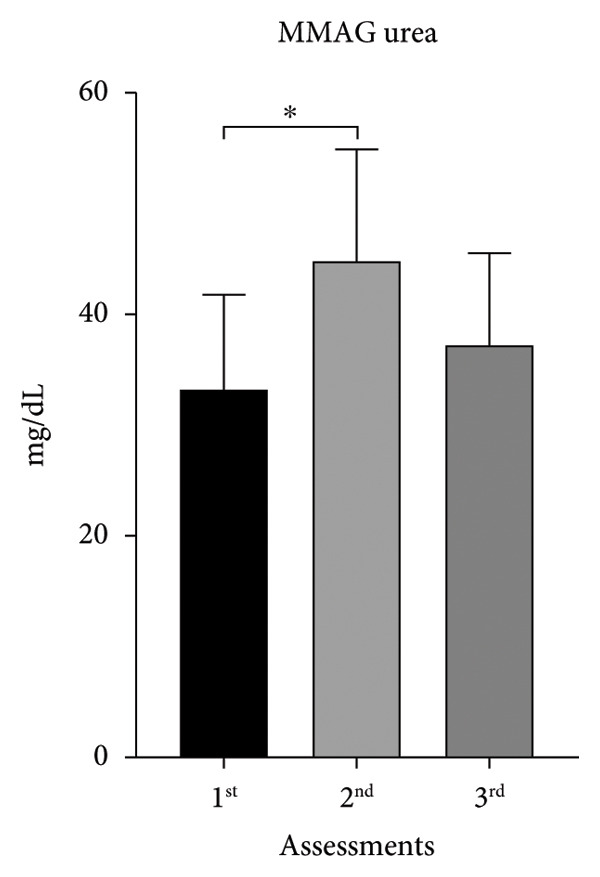
(a)

**Figure:**
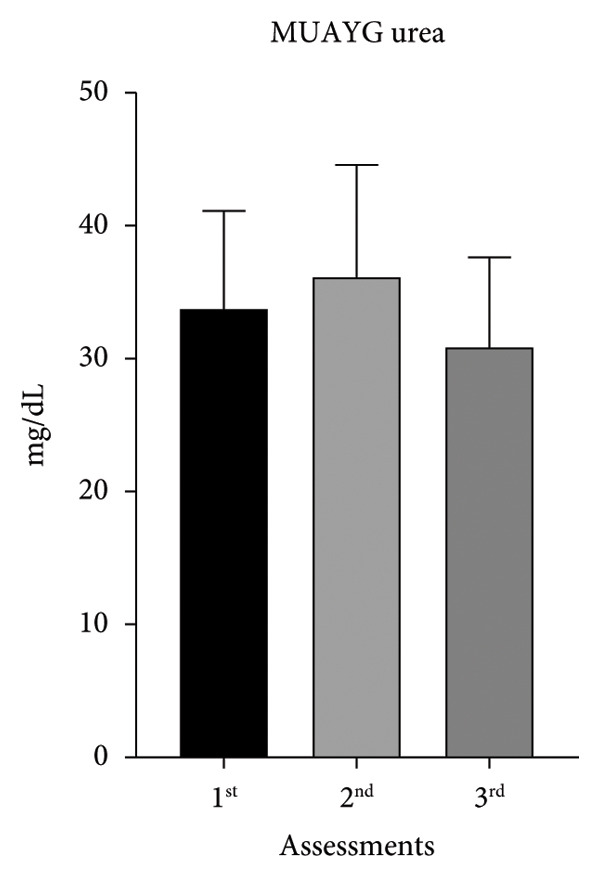
(b)

**Table 2:** Correlation among body weight, blood creatinine, blood urea, glomerular filtration rate (GFR) and proteinuria in the three evaluations for mixed martial.

	MMAG			MUAYG		
	Assessments					
	1^st^	2^nd^	3^rd^	1^st^	2^nd^	3^rd^
Body weight (kg)	74.7 ± 6.6	67.3 ± 8.4	73.3 ± 7.7	70.6 ± 8.3	64.9 ± 6.4	69.8 ± 7.1
Blood creatinine (mg/dL)	1.08 ± 0.08	1.31 ± 0.19^#^	1.09 ± 0.96^&^	1.10 ± 0.17	1.20 ± 0.11	1.12 ± 0.23
Blood urea (mg/dL)	33.13 ± 8.50	44.80 ± 10.05^Δ^	37.13 ± 8.33^β^	33.60 ± 7.42	36.13 ± 8.38	30.80 ± 6.71
GFR (mL/min/1.73 m^2^)	97.7 ± 8.9	79.1 ± 15.6^∗^	96.5 ± 10.5^∗∗^	97.2 ± 15.1	87.2 ± 10.6^∗^	102.2 ± 17.5^∗^
Presence of proteinuria (%)	53	73	40	60	100	13
Presence of hematuria (%)	0	6.7	6.7	6.7	0	20
Presence of nitrites (%)	0	26.7	13.3	0	0	13.3
Presence of leukocyturia (%)	0	46.7	6.7	0	26.7	0

*Note:* Water loss promoted significant changes in kidney function, as assessed by GFR estimated using the CKD‐EPI 2021 equation, in the GMMA group (1^st^ vs 2^nd^ assessments; 2^nd^ vs 3^rd^ assessments; ^∗^ *p* < 0.05) and in the GMUAY group (2nd vs 3^rd^ assessments;^∗∗^ *p* < 0.05); in creatinine in the GMMA group (1^st^ vs 2^nd^ assessments: ^#^ *p* < 0.05; 2^nd^ vs 3^rd^ assessments: ^&^ *p* < 0.05); and in urea the GMUAY group (1^st^ vs 2^nd^ assessments: ^Δ^ *p* < 0.05; 2^nd^ vs 3^rd^ assessments: ^β^ *p* < 0.05). Data are presented as mean ± SD. Repeated measures ANOVA followed by post hoc testing was performed, with a significance level set at 5%.

Also, as shown in Table 2, glomerular filtration rate (GFR) levels varied inversely proportional to urea and creatinine, reaching their lowest scores on the second assessments (MMAG: 79.1 mL/min/1.73 m^2^; MUAYG: 87.2 mL/min/1.73 m^2^). The peak decrease in GFR at the second assessment suggests transient kidney impairment at the height of dehydration.

## 5. Discussion

To ensure fairness in competition, athletes are classified into weight categories, creating a more balanced contest in terms of strength, agility, and body size [8].

However, to enter an inferior category, many athletes reduce their BW before the competition. Historically, athletes in combat sports have used aggressive methods to lose weight before competitions, with rapid weight loss being a common strategy. The primary aim is to lose body weight to qualify for a lower weight class, allowing the fighter to face an opponent with a weight disadvantage. Common weight loss techniques include restriction of food and liquid intake, excessive use of saunas and plastic clothing, inducing diuresis and vomiting, and the use of diuretics and laxatives, although the latter is prohibited by the World Anti‐Doping Agency [8]. Not all competitions follow these recommendations and prohibitions.

Dehydration, used as a method for weight reduction, can lead to a series of physiological alterations that may be detrimental to an athlete’s health. Park and colleagues [24] have shown that dehydration can reduce blood volume, plasma volume, and systolic arterial pressure. It also impacts heat dissipation, which is critical for maintaining body temperature during intense workouts. Dehydration increases plasma osmolarity, blood viscosity, and concentrations of various substances, such as creatinine, urea, cortisol, and ammonia in the blood. These changes can result in increased catecholamine responses, contributing to further physiological strain [9, 24, 25].

In this study, involving MMAG and MUAYG athletes, the average age of participants was 25 years old, like other studies on rapid weight loss in combat athletes, such as Jetton and colleagues (2013) (average age: 25.2) [26], Barley and colleagues (2018) (average age: 23.4) [27], and Cannataro and colleagues (2020) (average age: 25.8) [28] indicating that the age range of athletes participating in weight loss practices is relatively consistent, with most athletes being in their mid‐20s.

It was also found that athletes in both MMAG and MUAYG had many lengths of experience, ranging from 2 to 3 years of practice, similar to a study by Del Vecchio and Ferreira on conditioning routines and physical fitness in Pelotas, Brazil, where it was noted that 37.5% of participants had been practicing for at least 1.5 years, 37.5% for 4–6 years, and 25% for more than 8 years [29].

Weight manipulation is a well‐known practice in combat sports, being used by 80% and 100% of MMAG and MUAYG, respectively. In this study, similar to the profile presented by Connor and Egan in their study with professional and amateur MMA athletes, 97% of them stated that they used weight loss methods during the competition periods [30]. However, it is important to recognize that these practices often lead to negative health outcomes. It is believed that the tactical strategy of reducing body weight to enter a lower weight class than one’s own, and then rapidly gaining weight after the official weigh‐in, may provide some advantage during the fight. Physiologically, these practices can potentially contribute to the risk of injury, health problems, and even death [30].

Brechny et al. investigated the strategies used by MMA athletes to lose weight rapidly, identifying severe calorie restriction, water loading (excessive fluid intake followed by fluid restriction), active dehydration through sweat‐inducing activities, and the use of diuretics and laxatives as common methods [31]. Coswig et al. also observed that the most common techniques for rapid weight loss in combat sports include increasing exercise intensity, restricting food and liquid intake, using plastic suits, visiting saunas, and taking laxatives and diuretics [32]. These results are similar to the ones found in this study, demonstrating that the use of these techniques is widely practiced.

As a result of the use of weight loss techniques, the evaluation of volunteers’ arterial pressure and HR in the present study revealed significant changes between the various phases investigated, differing from the results observed by Lee et al. in Taekwondo athletes [33]. Chemical urine analysis using polyelectrolyte strips, as described by Glissmeyer et al., can be an essential tool for monitoring kidney health and identifying potential urinary tract diseases or other systemic conditions [34]. MMAG urine samples from the first assessments did not show the presence of hematuria, urobilinogen, nitrites, ketonic bodies, or leukocytes, but bilirubin (54%), proteinuria (53%), and glucosuria (13%) were detected. In contrast, MUAYG showed glucosuria (20%), hematuria (7%), nitrites (27%), bilirubin (53%), proteinuria (74%), ketonuria (40%), and leukocyturia (47%). The second assessments showed changes in both groups. In the MMAG, there was an increase in proteinuria (73%), while nitrites, ketone bodies, leukocytes, and glucosuria were detected in some samples. For MUAYG, hematuria, urobilinogen, and nitrites were absent, but 67% of the samples tested positive for bilirubin, 13% for glucose and ketonic bodies, and 27% for leukocytes. These findings indicate a significant difference in the physiological profiles between the two groups, similar to the results described by Glissmeyer et al. These results reflect the altered metabolic state of the athletes as they approach the weigh‐in.

On the third assessments, MMAG urine samples were positive for proteinuria, bilirubin, glucose, and leukocytes, while MUAYG exhibited a reduction in proteinuria and glucosuria, but some results were positive for hematuria and nitrites. These variations in urine biomarkers are indicative of the physiological stress caused by rapid weight loss and subsequent rehydration strategies.

Several studies have suggested that changes in kidney function, particularly blood creatinine levels, are important indicators of the impact of rapid weight loss on kidney health. There was a change in creatinine on the day of the fight and the day of the official weigh‐in, suggesting a change in creatinine clearance, which was elevated during all three observation periods. Previous studies have shown that creatinine levels are closely linked to muscle metabolism, and in athletes, higher creatinine levels are often observed due to increased muscle mass and activity [25, 35].

While they may not have had a statistical correlation with the small sample size, this study still has marked significance as it highlights the impact of deliberate dehydration on kidney function. Importantly, note that the GFRs do not fully return to baseline by the third assessment.

## 6. Conclusion

The profile of the athletes evaluated in this study is consistent with that reported in the literature when correlating mean age, body weight, and the most commonly adopted methods for rapid weight loss before the official weigh‐in. It was observed that food and fluid restriction, combined with activities that stimulate sweating, are the most frequently employed strategies by these fighters. In this study, elevated creatinine clearance was found, suggesting kidney damage associated with decreased glomerular filtration due to dehydration. This finding highlights the need to guide these athletes towards training and weight reduction methods that are less harmful to their physical integrity, since the radical measures commonly adopted for rapid weight loss are often implemented without due consideration of physiological homeostasis. Such practices may result in health consequences, including the onset of AKI, which, if left untreated, can progress to chronic kidney disease and lead to adverse effects in other organ systems. This article provides original data on the impacts that combat athletes can suffer during weight loss, providing solid evidence regarding the health consequences for athletes and the need to develop safe techniques for weight management.

## Conflicts of Interest

The authors declare no conflicts of interest.

## Funding

This study was supported by São Paulo Research Foundation (FAPESP, processes 2018/16653‐7 and 2017/17027‐0) and by the Coordenação de Aperfeiçoamento de Pessoal de Nível Superior ‐ Brasil (CAPES).

## Supporting Information

The Supporting information included refers to the previously developed questionnaire designed to investigate important clinical characteristics of each athlete’s profile included in the sample. This includes personal characteristics, the specific sport, and their practices related to weight control and dehydration.

## Supporting information


**Supporting Information** Additional supporting information can be found online in the Supporting Information section.

## Data Availability

The data for this manuscript are contained in the repository of the University where the thesis was deposited as per the link https://repositorio.unifesp.br/items/ba7b0de4-0ceb-4463-937e-2c81cbffb793, which is publicly accessible.

## References

[1] Verghese D. , Essential Ju-Jitsu: Origins, Principles & Practices, 2022, 1st edition, Dan Verghese.

[2] Bounty P. L. , Campbell B. I. , Galvan E. , Cooke M. , and Antonio J. , Strength and Conditioning Considerations for Mixed Martial Arts, Strength and Conditioning Journal. (2011) 33, no. 1, 56–67, 10.1519/SSC.0b013e3182044304, 2-s2.0-79955966802.

[3] Oliveira e Silva J. and Gagliardo L. , Análise Sobre Os Métodos e Estratégias de Perda de Peso Em Atletas de MMA No Período Pré Competitivo, RBNE-Revista Brasileira de Nutrição Esportiva. (2014) 8, no. 43.

[4] Paiva L. , Pronto Pra Guerra: Preparação Física Específica Para Luta and Superação, 2008, Omp Editora.

[5] Vasques D. , As Artes Marciais Mistas (MMA) Como Esporte Modeno: Entre a Busca Da Excitação e a Tolerância À Violência, Rev Esporte e Sociedade. (2013) 22, no. 8.

[6] Awi F. , Filho Teu Não Foge a Luta: Como Os Lutadores Brasileiros Transformaram O MMA Em Um Fenômeno Mundial, 2012, Intrinseca.

[7] Valente B. and Silva R. , Avaliar Os Efeitos da Desidratação Para Os Atletas de MMA (Mixed Martial Arts) e a Existência de Métodos Mais Eficientes, Anais do EVINCI. (2016) 2, no. 1.

[8] Franchini E. , Brito C. J. , and Artioli G. G. , Weight Loss in Combat Sports: Physiological, Psychological and Performance Effects, Journal of the International Society of Sports Nutrition. (2012) 9, no. 1, 10.1186/1550-2783-9-52, 2-s2.0-84870888985. PMC3607973 23237303

[9] Barley O. R. , Iredale F. , Chapman D. W. , Hopper A. , and Abbiss C. R. , Repeat Effort Performance is Reduced 24 hours After Acute Dehydration in Mixed Martial Arts Athletes, Journal of Strength & Conditioning Research. (2018) 32, no. 9, 2555–2561, 10.1519/JSC.0000000000002249, 2-s2.0-85052368925. 28930879

[10] Reale R. , Slater G. , and Burke L. M. , Individualised Dietary Strategies for Olympic Combat Sports: Acute Weight Loss, Recovery and Competition Nutrition, European Journal of Sport Science. (2017) 17, no. 6, 727–740, 10.1080/17461391.2017.1297489, 2-s2.0-85015624725. 28316263

[11] Rossi L. , Reis V. , and Azevedo C. , Desidratação e Recomendações Para a Reposição Hídrica Em Crianças Fisicamente Ativas, Revista Paulista de Pediatria. (2010) 28, no. 3, 337–345, 10.1590/s0103-05822010000300013, 2-s2.0-78149481865.

[12] Caldwell J. E. , Ahonen E. , and Nousiainen U. , Differential Effects of Sauna-Diuretic-And Exercise-Induced Hypohydration, Journal of Applied Physiology. (1984) 57, no. 4, 1018–1023, 10.1152/jappl.1984.57.4.1018. 6501022

[13] Trangmar S. J. and González-Alonso J. , Heat, Hydration and the Human Brain, Heart and Skeletal Muscles, Sports Medicine. (2019) 49, no. S1, 69–85, 10.1007/s40279-018-1033-y, 2-s2.0-85060568633. 30671905 PMC6445826

[14] Crighton B. , Close G. L. , and Morton J. P. , Alarming Weight Cutting Behaviours in Mixed Martial Arts: A Cause for Concern and a Call for Action, British Journal of Sports Medicine. (2016) 50, no. 8, 446–447, 10.1136/bjsports-2015-094732, 2-s2.0-84962815786. 26459278

[15] Lakicevic N. , Paoli A. , Roklicer R. et al., Effects of Rapid Weight Loss on Kidney Function in Combat Sport Athletes, Effects of Rapid Weight Loss on Kidney Function in Combat Sport Athletes. Medicina. (2021) 57, no. 6, 10.3390/medicina57060551. PMC8229569 34072641

[16] Miguel G. , A Importância Da Hidratação Em Esportes Coletivos, 2018, Universidade Federal de Pernambuco.

[17] Artioli G. G. , Saunders B. , Iglesias R. T. , and Franchini E. , It is Time to Ban Rapid Weight Loss from Combat Sports, Sports Medicine. (2016) 46, no. 11, 1579–1584, 10.1007/s40279-016-0541-x, 2-s2.0-84964403864. 27102173

[18] Reis V. , Seelaender M. , and Rossi L. , Impacto da Desidratação Na Geração de Força de Atletas de Arco e Flecha Durante Competição Indoor e Outdoor, Revista Brasileira de Medicina do Esporte. (2010) 16, no. 6.

[19] Kasper A. M. , Crighton B. , Langan-Evans C. et al., Case Study: Extreme Weight Making Causes Relative Energy Deficiency, Dehydration and Acute Kidney Injury in a Male Mixed Martial Arts Athlete, International Journal of Sport Nutrition and Exercise Metabolism. (2019) 29, no. 3, 331–338, 10.1123/ijsnem.2018-0029, 2-s2.0-85065592451. 29989458

[20] Hohenstein C. , Schmidt-Loucks J. , and Haefner H. , Dehydration and Its Effect on Acute Kidney Injury: A Review, Current Opinion in Nephrology and Hypertension. (2020) 29, no. 3, 257–263, 10.1097/MNH.0000000000000592.

[21] Koza Y. , Acute Kidney Injury: Current Concepts and New Insights, Journal of Injury and Violence Research. (2014) 8, no. 1, 58–62, 10.5249/jivr.v8i1.610. PMC4729334 26804946

[22] Reina-Couto M. , Afonso J. , Carvalho J. et al., Interrelationship Between Renin-Angiotensin-Aldosterone System and Oxidative Stress in Chronic Heart Failure Patients With or Without Renal Impairment, Biomedicine and Pharmacotherapy. (2021) 133, 10.1016/j.biopha.2020.110938. 33171402

[23] Popkin B. M. , D’Anci K. E. , and Rosenberg I. H. , Water, Hydration, and Health, Nutrition Reviews. (2010) 68, no. 8, 439–458, 10.1111/j.1753-4887.2010.00304. 20646222 PMC2908954

[24] Park S. , Alencar M. , Sassone J. , Madrigal L. , and Ede A. , Self-Reported Methods of Weight Cutting in Professional Mixed-Martial Artists: How Much are They Losing and Who Is Advising Them?, Journal of the International Society of Sports Nutrition. (2019) 16, no. 1, 10.1186/s12970-019-0320-9. PMC6849211 31718652

[25] Banfi G. and Del Fabbro M. , Relation Between Serum Creatinine and Body Mass Index in Elite Athletes of Different Sport Disciplines, British Journal of Sports Medicine. (2006) 40, no. 8, 675–678, 10.1136/bjsm.2006.026658, 2-s2.0-33746925227. 16723402 PMC2579448

[26] Jetton A. M. , Lawrence M. M. , Meucci M. et al., Dehydration and Acute Weight Gain in Mixed Martial Arts Fighters Before Competition, Journal of Strength and Conditioning Research. (2013) 27, no. 5, 1322–1326, 10.1519/JSC.0b013e31828a1e91, 2-s2.0-84878091206. 23439336

[27] Barley O. R. , Chapman D. W. , and Abbiss C. R. , Weight Loss Strategies in Combat Sports and Concerning Habits in Mixed Martial Arts, International Journal of Sports Physiology and Performance. (2018) 13, no. 7, 933–939, 10.1123/ijspp.2017-0715, 2-s2.0-85052387962. 29283792

[28] Cannataro R. , Cione E. , Gallelli L. , Marzullo N. , and Bonilla D. A. , Acute Effects of Supervised Making Weight on Health Markers, Hormones and Body Composition in Muay Thai Fighters, Sports. (2020) 8, no. 10, 10.3390/sports8100137. PMC7602705 33081214

[29] Del Vecchio F. and Ferreira J. , Mixed Martial Arts: Rotinas de Condicionamento e Avaliação da Aptidão Física de Lutadores de Pelotas/RS1, Revista Brasileira de Ciências do Esporte. (2013) 35, no. 3, 611–626, 10.1590/s0101-32892013000300007, 2-s2.0-84892590496.

[30] Connor J. and Egan B. , Prevalence, Magnitude and Methods of Rapid Weight Loss Reported by Male Mixed Martial Arts Athletes in Ireland, Sports. (2019) 7, no. 9, 10.3390/sports7090206. PMC6783947 31505745

[31] Brechney G. C. , Chia E. , and Moreland A. T. , Weight-Cutting Implications for Competition Outcomes in Mixed Martial Arts Cage Fighting, Journal of Strength and Conditioning Research. (2021) 35, no. 12, 3420–3424, 10.1519/JSC.0000000000003368. 31567789

[32] Coswig V. S. , Miarka B. , Pires D. A. , Da Silva L. M. , Bartel C. , and Del Vecchio F. B. , Weight Regain, but Not Weight Loss, is Related to Competitive Success in Real-Life Mixed Martial Arts Competition, International Journal of Sport Nutrition and Exercise Metabolism. (2019) 29, no. 1, 1–8, 10.1123/ijsnem.2018-0034, 2-s2.0-85060135586. 29757051

[33] Lee S. H. , Pekas E. J. , Lee S. , Headid R. J. , and Park S. Y. , The Impact of Aspirin Intake on Lactate Dehydrogenase, Arterial Stiffness, and Oxidative Stress During High‐Intensity Exercise: a Pilot Study, Journal of Human Kinetics. (2020) 72, no. 1, 101–113, 10.2478/hukin-2019-0101. 32269652 PMC7126265

[34] Glissmeyer E. W. , Korgenski E. K. , Wilkes J. et al., Dipstick Screening for Urinary Tract Infection in Febrile Infants, Pediatrics. (2014) 133, no. 5, e1121–e1127, 10.1542/peds.2013-3291, 2-s2.0-84899797523. 24777232 PMC4006440

[35] Banfi G. , Del Fabbro M. , and Lippi G. , Serum Creatinine Concentration and Creatinine-Based Estimation of Glomerular Filtration Rate in Athletes, Sports Medicine. (2009) 39, no. 4, 331–337, 10.2165/00007256-200939040-00005, 2-s2.0-63149188302. 19317520

