# Supplementary material for: Acute Kidney Injury Associated With Dehydration Protocol Used by Combat Sports Athletes
Source: Transl Sports Med. 2026 Feb 2;2026:2946676. doi: 10.1155/tsm2/2946676 (PMC12863454; doi:10.1155/tsm2/2946676)
Supplement: Supplementary file 1 — Supporting Information Additional supporting information can be found online in the Supporting Information section. [file TSM2-2026-2946676-s001.docx]

**QUESTIONÁRIO ELABORADO/ELABORATED QUESTIONNAIRE**

***Perfil do Atleta/Athlete’s Profile***

N⁰ da amostra/Sample number:______

**GMUAY ( )/ GMMA( )**

Data/Date:____/_____/________

**Nome/Name:___________________________________________________**

**Data de Nascimento/Date of Birth:_______________ Idade/Age:_____**

**Sexo/Sex:_________**

1. **Qual sua modalidade de base?/What is your base modality?**

( ) Jiu Jitsu; ( ) Muay Thai; ( ) Judô/Judo; ( ) Capoeira; ( ) MMA;

( ) Outras/Others:________________________________________

1. **Há quanto prática qualquer modalidade de lutas?/For how long have you been practicing any fighting modality?**

( ) Menos de dois anos/Less than two years; ( ) 2 à 4 anos/2 to 4 years; ( ) 5 à 10 anos/5 to 10 years; ( ) Mais de 10 anos/More than 10 years.

1. **Qual seu peso corporal médio e respectiva categoria relacionada ao peso da sua modalidade de base?/What are your mean body weight and respective weight-related category in your base modality?**

Peso corporal/Body weight:______Kg; Categoria/Category:________________________

1. **Qual (s) sua (s) graduação (s)? *Exemplo: Faixa Preta, Cordão verde e amarelo dentre outras./What are your graduations? E.g.: Black belt, Green and yellow belt, among others.***

Jiu Jitsu: ____________________ Muay Thai: ____________________

Judô/Judo: _______________________ Capoeira ______________________

Outras/Others:___________________________________________________

1. **Há quanto pratica lutas?/For how long have you been practing fighting?**

( ) Menos de dois anos/Less than two years; ( ) 2 à 4 anos/2 to 4 years; ( ) 5 à 10 anos/5 to 10 years; ( ) Mais de 10 anos/More than 10 years.

1. **Participa de eventos competitivos de lutas? Há quanto tempo?/Do you participate in competitive fight events? For how long?**

( ) Não/No; ( ) Sim/Yes: ( ) Menos de dois anos/Less than two years; ( ) 2 à 4 anos/2 to 4 years; ( ) 5 à 10 anos/5 to 10 years; ( ) Mais de 10 anos/More than 10 years.

1. **Quantos eventos de lutas você participou?/In how many fight events have you participated?**

( ) 0; ( ) 1; ( )2; ( ) 3 – 5; ( ) 6 – 7; ( ) 8 – 10; ( ) Mais que 10/More than 10 years.

1. **Costuma perder peso para lutar? Quantos quilos em média você perde para lutar?/Do you normally lose weight to fight? How much weight do you normally lose before Fighting?**

( ) Não/No; ( ) Sim/Yes: ( ) 1 – 2kg; ( ) 3 – 5kg; ( ) 7 – 10kg; ( ) 11– 15kg; ( ) Mais que 15kg/More than 15kg.

1. **Qual o tempo médio utilizado para perda do peso corporal antes do evento?/ What is the average time it takes you to lose body weight before the event?**

| Mais de 6 meses antes da pesagem oficial/More than 6 months before weighing. | ( ) | 20 – 29 dias antes da pesagem oficial/20-29 days before weighing. | ( ) |
| --- | --- | --- | --- |
| 5 – 6 meses antes da pesagem oficial/5-6 months before weighing. | ( ) | 15 – 19 dias antes da pesagem oficial/15-19 days before weighing. | ( ) |
| 3 – 4 meses antes da pesagem oficial/3-4 months before weighing. | ( ) | 10 – 14 dias antes da pesagem oficial/10-14 days before weighing. | ( ) |
| 2 meses antes da pesagem oficial/2 months before weighing. | ( ) | 7 – 9 dias antes da pesagem oficial/7-9 days before weighing. | ( ) |
| 1 mês antes da pesagem oficial/1 month before weighing. | ( ) | 5 – 6 dias antes da pesagem oficial/5-6 days before weighing. | ( ) |
| XXX | ( ) | 3 – 4 dias antes da pesagem oficial/3-4 dasy before weighing. | ( ) |
| XXX | ( ) | 1 – 2 dias antes da pesagem oficial/1-2 days before weighing. | ( ) |
| XXX | ( ) | No dia da pesagem oficial/on the weighing day. | ( ) |

1. **Você utiliza a desidratação como forma de redução do peso corporal? Através de quais meios?/Do you use dehydration as a menos of body weight reduction? Using which techniques?**

( ) Não/No;

( ) Sim/Yes:

( ) Sudorese induzida pela prática de atividades físicas/Sweating induced by physical activities;

( ) Exposição à sauna/ Sauna exposition;

( ) Realização de exercícios físicos vigorosos com roupas plásticas e/ou em ambientes quentes e úmidos/Performing vigorous physical exercising with plastic clothes and/or in hot and humid areas;

( ) Uso de diuréticos/Use of diuretics.

( ) Outros/Others:_______________________________________________________

1. **É portador de alguma das doenças abaixo?/Do you have any of the diseases below?**

( ) Não/No;

( ) Sim/Yes:

( ) Diabetes Mellitus tipo/type 1;

( ) Diabetes Mellitus tipo/type 2,

( ) Doenças Renais/Kidney diseases;

( ) Hipertensão Arterial/Blood hypertension;

( ) Cardiopatias/Heart diseases.

( ) Outros/Others:___________________________________________________________

1. **Possui antecedentes familiares portadores de alguma das doenças abaixo?/Do you have any family history on the diseases below?**

( ) Não/No;

( ) Sim/Yes:

( ) Diabetes Mellitus tipo/type 1;

( ) Diabetes Mellitus tipo/type 2,

( ) Doenças Renais/Kidney diseases;

( ) Hipertensão ArterialBlood hypertension;

( ) Cardiopatias/Heart diseases.

( ) Outros/Others:_______________________________________________________
